# Enhanced Follicular Dendritic Cell-B Cell Interaction in HIV and SIV Infections and its Potential Role in Polyclonal B Cell Activation

**DOI:** 10.1155/1998/34014

**Published:** 1998

**Authors:** Yvonne J. Rosenberg, Mark. G. Lewis, Marie H. Kosco-Vilbois

**Affiliations:** 1 Therlmmune Inc. Rockville Maryland 20852 USA; 2 Henry M. Jackson Foundation Rockville Maryland 20850 USA; 3 Glaxo Institute for Molecular Biology Geneva Switzerland

## Abstract

Human immunodeficiency virus (HIV) infections have been characterized by both polyclonal Bcell activation and enhanced responsiveness to B-cell growth factors on one hand and the loss of specific antibody (Ab) responses and refractoriness to the normal signals for B-cell activation on the other. Histopathological studies of lymph node from HIV- and simian immunodeficiency virus (SIV)-infected individuals have indicated initial follicular hyperplasia and the appearance of large irregular germinal centers that undergo progressive involution concomitant with follicular dendritic-cell (FDC) disruption. During this process, follicular dendritic-cell -enriched lymph-node-cell cultures exhibit increased ability to induce cluster formation (“*in vitro* germinal centers”), lymphocyte proliferation and antibody production compared to uninfected controls. This paper discusses how enhanced FDC-B-cell interaction within SIV-infected germinal centers may result in a reduced ability to select high-affinity B cells and alter the dynamics of antibodyproducing- cell and memory-cell generation resulting in the observed hyperactivity.


*Developmental Immunology*, 1998, Vol. 6, pp. 61–70
Reprints available directly from the publisher
Photocopying permitted by license only

© 1998 OPA (Overseas Publishers Association)
N.V. Published by license under the
Harwood Academic Publishers imprint,
part of the Gordon and Breach Publishing Group
Printed in Malaysia

### Enhanced Follicular Dendritic Cell-B Cell Interaction in HIV and SIV Infections and Its Potential Role in Polyclonal B Cell Activation

YVONNE J. ROSENBERG[a*], MARK. G. LEWIS[b] and MARIE H. KOSCO-VILBOIS[c]

[a]*TherImmune Inc. Rockville, Maryland 20852*; [b]*Henry M. Jackson Foundation, Rockville, Maryland 20850, USA*; [c]*Glaxo Institute for Molecular Biology, Geneva, Switzerland*

*(Received 2 September 1996; In final form 30 May 1997)*

Human immunodeficiency virus (HIV) infections have been characterized by both polyclonal B-cell activation and enhanced responsiveness to B-cell growth factors on one hand and the loss of specific antibody (Ab) responses and refractoriness to the normal signals for B-cell activation on the other. Histopathological studies of lymph node from HIV- and simian immunodeficiency virus (SIV)-infected individuals have indicated initial follicular hyperplasia and the appearance of large irregular germinal centers that undergo progressive involution concomitant with follicular dendritic-cell (FDC) disruption. During this process, follicular dendritic-cell -enriched lymph-node-cell cultures exhibit increased ability to induce cluster formation ("*in vitro* germinal centers"), lymphocyte proliferation and antibody production compared to uninfected controls. This paper discusses how enhanced FDC-B-cell interaction within SIV-infected germinal centers may result in a reduced ability to select high-affinity B cells and alter the dynamics of antibody-producing-cell and memory-cell generation resulting in the observed hyperactivity.

### INTRODUCTION

In addition to the well-documented changes in the level and function of T cells, HIV infections also lead to dramatic qualitative and quantitative changes in the B cell compartment in terms of both function and the specificity of the antibody molecules produced. *In vivo* abnormalities in B-cell function include polyclonal B-cell activation, hypergammaglobulinaemia, increased levels of autoantibodies, decreased ability to mount antigen (Ag)-specific responses, and premature loss of specific anti-HIV antibodies (Zolla-Pazner, 1984; Mizuma et al., 1988; Amadori and Chieco-Bianchi, 1990; Fenouillet et al., 1992). Accordingly, cultured peripheral blood mononuclear cells (PBMC) from seropositive patients secrete antibodies to HIV- and non-HIV-related antigens, spontaneously or following culture with HIV, and interleukins (IL), and exhibit decreased responsiveness to mitogens (Amadori et al., 1989; Edelman and

*Corresponding author.

Zolla-Pazner, 1991; Delfraissy et al., 1992; Shirai et al., 1992). Although the observed polyclonal B-cell activation occurs early and persists throughout infection, it is thought to be independent of the specific humoral anti-HIV response, which clearly predominates during asymptomatic infection, as evidenced by large number of B cells spontaneously secreting anti-HIV antibodies in patient blood (Amadori et al., 1989; Shirai et al., 1992), bone marrow (BM) (Cosentino et al., 1994), and duodenum (Eriksson et al., 1995). In addition, antibody-secreting cells (ASC) specific for HIV proteins and peptides have also been detected around follicles and in the medulla in HIV+LN biopsies (Laman et al., 1989). *In vitro*, the generation of HIV− and cytokine− (e.g., IL-6, IL-2 plus interferon-$\alpha$ [IFN-$\alpha$]) induced anti-HIV ASC in PBMC cultures derived from HIV-seropositive but not -seronegative individuals further illustrates extensive *in vivo* activation and expansion of HIV-specific B cells (Amadori et al., 1991; Delfraissy et al., 1992; Chirmule et al., 1993).

During the course of disease, which varies amongst individuals, the number of anti-HIV ASC in blood and the levels of serum antibody specific for particular HIV determinants decline. Although the loss of certain specificities, for example, anti-p24, has been associated with disease progression, the duration of specific responses usually has not been shown to correlate with clinical, immunological, or virologic parameters (Yarchoan et al., 1986; Shirai et al., 1992; Binley et al., 1997). In contrast, the increase in polyclonal B-cell activation has been shown to have statistical significance among patients with advanced disease. Thus, in studies using PBMC by Shirai et al. (1992), high HIV envelope gp160- and gag p24-specific B-cell activation decreased, whereas polyclonal B-cell activation, as measured by the appearance of B-cell secreting anti-TNP and anti-DNA IgG antibodies increased during late-stage disease (CDC class IV). Likewise, the *in vitro* generation of HIV-ASC from PBMC from late-stage patients was also greatly reduced (Delfraissy et al., 1992). How this transition relates to the process by which ASC are generated and selected from B-cell precursors in lymphoid organs and how this loss in ASC reflects similar changes in tissues are critical to our understanding of AIDS pathogenesis.

In this context, histopathological studies on HIV/SIV organs have revealed dramatic changes in the follicles (B-cell area) of lymphoid organs that represent a dynamic process progressing from follicular hyperplasia to follicular involution (Biberfeld et al., 1986; Racz et al., 1986; Vago et al., 1989; Persidsky et al., 1995). During this time, follicular dendritic cells (FDC), which normally create the environment in germinal centers (GC) for B cell somatic mutation, affinity maturation, and isotype switch following exposure to antigen (Kraal et al., 1982; Jacob et al., 1991; Liu et al., 1992; Apel and Berek, 1996; Liu and Banchereau, 1996), also themselves undergo initial hypertrophy and subsequent fragmentation of their reticular networks. Although these alterations are not specific for HIV/SIV infections (Janossy et al., 1991; Rideout et al., 1992), they are highly characteristic of HIV/SIV-related lymphadenopathy and are closely associated with both increased trapping of viral antigens by FDC and infiltration into the GC of CD45RAlo $CD8^+$ cells known to produce potentially lytic molecules. (Tenner-Racz et al., 1987; Vago et al., 1989; Devergne et al., 1991; Tenner-Racz et al., 1993; Rosenberg et al., 1994b). Since FDC are critical to the induction of specific ASC precursors and the generation and maintenance of B-cell memory (Klaus et al., 1980; MacLennan and Gray, 1986; Tew et al., 1992), the simplest assumption is that the B-cell abnormalities observed during infection directly result from virus-induced changes in the GC environment. For practical reasons, such studies cannot be easily performed in humans and our laboratory has therefore focused on examining the ability of mesenteric LN FDC derived from SIV-infected macaques to support GC formation, lymphocyte growth, and antibody formation *in vitro*. The findings demonstrated that such FDC have enhanced function when compared to FDC from control cultures. The purpose of this paper is to describe the possible mechanisms underlying the decline in certain specific anti-HIV/SIV titers and polyclonal expansion of B cells secreting non-HIV-related antibodies in the context of increased FDC function.

### RESULTS AND DISCUSSION

The ability of FDC derived from SIV-infected LN to induce cluster formation, activate and induce proliferation of B and $CD8^+$ cells, and to induce Ig and Ab production has been examined (Rosenberg et al., 1997). To this end, FDC-enriched mesenteric LN cell cultures from SIV-infected macaques were functionally compared to two controls: (1) FDC-enriched mesenteric LN cultures from uninfected macaques and (2) non-FDC-containing LN-cell suspensions derived from the same LN. The findings indicated:

1. Greatly enhanced spontaneous cluster formation in FDC-enriched LN cultures from SIV-infected macaques. In the absence of exogenous cytokines, such cultures contained many large clusters comprising thousands of cells in addition to smaller clusters of 10-100 cells containing predominantly B cells. By contrast, uninfected monkey donors contained mostly small clusters, although occasional large clusters were also observed in uninfected macaques, suggesting ongoing environmental stimulation leading to GC formation *in vivo*. The increased size of the clusters also corresponded with prolonged cell survival. For example, FDC-enriched cultures derived from the LN of the SIV-251-infected macaque 93 days post infections (pi) supported >40% B cells for 10 days compared to death of control cultures by 5-7 days. In the presence of IL-2, $CD8^+$ and $CD56^+$ cells were also increased.

2. Enhanced ability of FDC-enriched cultures from LN of chronically SIV-infected to induce lymphocyte proliferation. Theses studies indicated that at peak time points (usually days 2-3), FDC-enriched LN cultures from infected macaques consistently incorporated 3-10 times higher cpm than control non-FDC-containing cell suspensions prepared from the same LN or control FDC-enriched cells from LN of uninfected monkeys. The level of incorporation also appeared to be associated with the CD4/CD8 ratio of the LN and the degree to which GC were infiltrated with $CD8^+$ lymphocytes. It should be noted that the addition of SIV-251 to fresh LN cells *in vitro* did not result in cluster formation or proliferation and may actually suppress anti-CD3-induced proliferation (unpublished observations).

3. Exogenous IL-2 at the initiation of culture resulted in greatly elevated lymphocyte proliferation at D4 in the FDC-enriched population from SIV-positive LN. For example, the [$^3$H-TdR] incorporation in FDC-enriched cultures was five fold higher than that in control non-FDC-containing suspension cultures.

4. High background proliferation in the non-FDC-containing cell cultures, occasionally observed in tissues of SIV-infected macaques is rapidly lost in the absence of FDC and IL-2.

5. Increased production of IgG1 immunoglobulin and anti-SIV Ab by FDC-enriched cultures from SIV-infected LN. ELISA results indicate that, in addition to their requirement for B-cell growth, FDC play an important role in the high-level production of IgG1 and specific anti-SIV Ab by B cells from infected LN. No anti-SIV Ab was observed in either of two FDC-enriched LN preparations derived from uninfected monkeys. The high levels of Ab specific for SIV do indicate the commitment of GC in SIV-infected macaques toward the production of anti-SIV Ab.

6. FDC-dependent clusters predominantly contained activated CD45RAlo $CD8^+$ cells and $CD56^+$ cells. Unlike clusters from immunized mice or uninfected human tonsil (Koopman et al., 1991; Petrasch et al., 1991; Kosco et al., 1992) where $CD8^+$ cells are rarely found, FDC-enriched cultures from infected macaques maintained 10-20% $CD8^+$ cells that were predominantly CD45RAlo. In keeping with this activated phenotype, the addition of IL-2 to cultures at day 12 appeared to further select for the expansion of $CD8^+$ cells. In control cultures, $CD8^+$ cells were almost totally CD456RAhi.

### GC Formation and HIV/SIV Infections

High-affinity IgG and IgA antibody synthesis and the generation of memory B cells involves complex cellular interactions among FDC, B cells, and T cells at several levels (Klaus et al., 1980; MacLennan and Gray, 1986; Tew et al., 1992). FDC, which initially "present" unprocessed Ab-complexed antigen to B

cells in association with the adhesion molecular pairs lymphocyte function-associated antigen-1 (LFA-1) — intercellular adhesion molecule-1 (ICAM-1) and very late antigen-4 (VLA-4) — vascular adhesion molecule-1 (VLA-1) (Koopman et al., 1991; Kosco et al., 1992). They also play a subsequent role in selecting the high-affinity mutants from among the pool of rapidly dividing centrocytes. Each of these FDC-B-cell interactions involves the delivery of a costimulatory signal(s) from the FDC to the B cell in addition to the antigen-specific interaction (Burton et al., 1993; Kosco-Vilbois et al., 1993). Although not yet identified, possible candidates for the costimulatory signals include the ligand for CD19, complement fragments on the FDC surface, other interactions involving CD23, adhesion molecules or chemokines. The $CD40^+$ B7 high B cells thus selected then present processed antigen to GC T cells inducing CD40 ligand (CD401) expression and the synthesis of cytokines such as IL-4 and IL-10, required for further B-cell proliferation, isotype switching, and Ab secretion. Normally, any B cell that expresses low-affinity Ig receptors or receptors specific for autoantigens do not normally receive the appropriate signals for further maturation, undergo programmed cell death (PCD) (Liu et al., 1991), and are taken up by tingible body macrophages.

Following HIV/SIV infections, virus-induced changes occur in LN GC that may enhance normal FDC-B cell interactions leading to polyclonal B cell activations and relative decreases in humoral HIV/SIV specific responses. During the period of follicular hyperplasia, GC increase in size and become irregular in shape and exhibit increased expression of adhesion molecules. The expanded area within the dark zone shows greatly increased FDC expression of CD23, rapidly proliferating $KI\text{-}67^+$ centroblasts, and an accumulation of infiltrating $CD45RO^+$ $CD8^+$ cells. In addition the expansion of the light zone, where complexed viral antigens are deposited and $CD4^+$ T cells are found, results in a much reduced mantle zone (Janossy et al., 1991; Joling et al., 1992; Persidsky et al., 1995). It is not currently known how an increase in the volume of the reticular network and increased FDC function relates to quantitative increases in FDC number. FDC-dependent and T cell-dependent mechanisms that may account for this observed GC B-cell hyperactivity consistent with these immunohistological changes are described below and depicted in Figure 1.

### Increased Survival of Low-Affinity and Autoreactive B Cells

### (i) gp120 Cross-Linking of Ig Receptors on Low-Affinity B Cells

As HIV/SIV infections progress, virus particles become bound to FDC complement receptors (CR) either directly via C3b, iC3b, and C3d on the virus (Marschang et al., 1993; Thielens et al., 1993) or via antibody bridges. Studies in mice with the MAIDS virus (Masuda et al., 1995) indicate that during the infection, previously bound soluble nonviral antigens are also displaced, presumably because virions bearing C3 components dislodge and replace the preexisting Ag-Ab complexes from the complement receptors on FDC (CR1, CD2, and CR3). In this situation, presentation of FDC-bound virions expressing membrane gp120 molecules in a spacially ordered arrangement could induce sufficient cross-linking of B-cell Ig receptors to activate B cells. The increased avidity resulting from high levels of multipoint binding may thus overcome affinity considerations and result in the activation of cells with very low specificity for viral antigens, including some high-frequency autoreactive B cells. Such binding by B cells in the presence of costimulation signals from FDC might serve to prevent the downregulation of Bcl-2 and up regulation of CD95 (*fas*) (Garrone et al., 1995) expression and render the cells susceptible to T-cell-derived signals, rescuing them from PCD that would normally have been their fate. Several predictions can be made according to this scenario: (1) Initially, depending on the B-cell repertoire of the individual, less stringent selection might result in an overall decrease in the percentage of total effective anti-HIV ASC, whereas that for other B-cell specificities might increase; and (2) whereas anti-gp120 antibody responses would be maintained at relatively high levels, those specific for monomeric soluble proteins, would remain low or be lost due to displacement from the FDC or an inability

(Binley et al., 1997); and (3) in studies using duodenal biopsies from AIDS patients, a marked decrease in the numbers of ASC specific for the soluble antigens keyhole limpet hemocyanin and dog serum albumin in the absence of any decline in the percent anti-gp160 ASC (Eriksson et al., 1995).

### (ii) Increased Expression of Adhesion and Activation Molecules on B Cells and FDC

Cytokines play a critical role in mediating immune function; the pattern of cytokine production determining the type of immune response generated. In addition, cytokines such as IFN-$\gamma$ and tumour necrosis factor-$\alpha$ (TNF-$\alpha$) are known to induce chemokines (Ebnet et al., 1996) and regulate lymphocyte migration and sequestration by virtue of their ability to induce adhesion molecules, eg, ICAM-1, VCAM-1, LFA-1 on lymphocytes, antigen-presenting cells, and vascular endothelium (Anderson and Reynolds, 1979; Mackay, 1991; Colditz and Watson, 1992; May and Ager, 1992; Adams et al., 1994). Since the ability of FDC-enriched LN cells to form clusters *in vitro* is known to be dependent on LFA-1-ICAM-1 and VLA-4-VCAM-1 interactions (Koopman et al., 1991; Kosco et al., 1992), it is highly likely that $CD8^+$ cell-derived IFN-$\gamma$ production in GC (Emilie et al., 1990; Graziosi et al., 1994) (unpublished observation) can induce increased expression of ICAM-1 and VCAM-1 on FDC and facilitate their binding to B cells. Enhanced VCAM-1 expression has been demonstrated in reactive human LN (Ruco et al., 1992) and more recently on FDC in GC of SIV-infected LN (Persidsky et al., 1995). Although increases in cells producing IL-1, IL-4, IL-6, TNF-$\alpha$, and IFN-$\gamma$ have been reported in LN and blood from HIV and SIV-infected individuals (Emilie et al., 1990; Fan et al., 1993; Graziosi et al., 1994; Rosenberg et al., 1994a; Persidsky et al., 1995), a comparison with the appropriate control LN or blood from uninfected monkeys or from humans with HIV-unrelated hyperplasia has indicated to date that only IFN-$\gamma$ production is consistently increased throughout infection.

Another alteration observed in GC of HIV-infected LN is the greatly increased expression of CD23 antigen in the expanded part of the "dark" zone: CD23 being expressed normally only on FDC and B cells in the apical light zone. This CD23 positivity, which may result from IL-4 synthesis or increased numbers of immune complexes or FDC (Maeda et al., 1992) may increase signalling to B cells via interaction with CD21 (Bonnefoy et al., 1993) and may skew the maturation of B cells into plasma cells rather than memory cells (Liu and Banchereau, 1996). Regardless of whether binding of integrins with their ligands or CD23 with CD21 is able to generate maturation signals *in vivo* (Liu et al., 1992; Bonnefoy et al., 1993; Adams et al., 1994; Lub et al., 1995; Liu and Banchereau, 1996), it is possible that such interactions may augment low-affinity binding of FDC-bound antigen to membrane Ig sufficient to allow the costimulation and/or differentiation of low-affinity B cells that would normally have been destined to die.

### Increase in T-Cell-Derived Maturation and Differentiation Signals

Maturation and differentiation of activated B cells into IgG and IgA ASC or memory cells require the participation of T cells either via cognate CD40-CD40L or B7-CD28 interactions and the elaboration of cytokines eg. IL-4 and IL-10 (Liu and Banchereau, 1996), or via nonspecific T-cell-derived signals, eg., TNF-$\alpha$ and IL-2 (Macchia et al., 1993). In the former case, LN GC from HIV+ individuals have been shown to have increased expression of CD40 ligand (Janossy, personal communication). In addition, although different studies have published variable findings with respect to IL-10 secretion/expression (Montaner, 1994), the evidence to date shows no obvious reduction in IL-10 levels in LN or blood during HIV infection. The high levels of IgG produced during HIV/SIV infections indicate that sufficient T-cell help is present to induce isotype switching.

In the case of nonspecific signaling, TNF-$\alpha$, produced by $CD4^+$ cells from HIV patients or infected cells *in vitro*, is able to induce IgG production by syngeneic (presumably activated) B cells (Macchia et al., 1993). In this way, any B cell of varying affinity for HIV antigens that are rescued by interaction with HIV-coated FDC and express TNF receptors 1 or 2 (TNFR-1 or 2) may be induced to secrete Ig. The TNF-$\alpha$ and IL-6 families of molecules are important at several levels. Thus, in addition to the critical role of TNF-$\alpha$, lymphotoxin-$\alpha$ (LT-$\alpha$), and TNFR-1 on FDC generation, GC formation and the levels of IgG production as recently demonstrated using knockout mice (Le Hir et al., 1996; Matsumoto et al., 1996), IL-6 is produced by FDC (M. K.-V., unpublished observation) and is important in spontaneous anti-HIV production by B cells (Amadori et al., 1991; Delfraissy et al., 1992). Furthermore TNF-$\alpha$ and IL-6 cytokines have also been closely linked with increased HIV and SIV replication (Birx et al., 1993; Scala et al., 1994; Virelizier, 1994), thereby increasing antigen levels.

Finally, it is noteworthy that whereas B cells constitute roughly 30% of total lymphocytes in the body, usually only 10-20% of the 1-2% of lymphocytes that circulate in the blood at any time are B cells (Westermann and Pabst, 1992). Thus the usual sampling of PBMC may not be an accurate means of assessing overall B-cell function. Accordingly, (1) the number of peripheral blood B cells secreting anti-gp-160 antibody shows no significant correlation with serum anti-HIV gp-160 antibody concentrations (Schwartz et al., 1994); (2) BM contains >30-fold more anti-gp160 ASC than blood following immunization with gp160 (Cosentino et al., 1994); and (3) levels of HIV-specific ASC in duodenal biopsies and BM are present in large numbers throughout infection when numbers in blood are very low (Eriksson et al., 1995). These results emphasize the need to examine tissues and not just blood in order to elucidate the mechanisms underlying the observed hyperactivity. In this context, another caveat arises because during the budding process, HIV virions become enveloped by the lipid bilayer of the host cell and simultaneously by many host-cell proteins (Arthur et al., 1992; Meerloo et al., 1993; Dierich, unpublished observation). The extent to which such molecules may be responsible for the increased expression of adhesion and activation molecules and receptors as well as enhanced effector function observed in HIV+ GC are currently only beginning to be understood.

### MATERIALS AND METHODS

### Animals and Infections

Rhesus macaques were infected with 1-10 tissue culture infectious doses 50 ($TCID_{50}$) of SIV-251 and sacrificed between days 93 and 535, at which time mesenteric LN were removed for assaying. Control LN were collected from rhesus and pig-tailed macaques (Regional Primate Center, Seattle, WA).

### Analysis of Lymphocyte Populations by Flow Cytometry (FCM)

Fresh or cultured cells were double or triple stained with fluorescein isothyiocyanate (FITC)-coupled CD45RA mAb (Gentrak, Wayne, PA), anti-CD4, and CD56 coupled to phycoerythrin (PE) and CD20 and CD8 mAbs coupled to either FITC or peridinin chlorophyll protein (PerCP; Becton Dickenson, Mountain View, CA). Analysis was done on a FACScan (Becton Dickenson).

### Isolation of Cells and Culture Conditions

FDC-enriched cultures were prepared using mesenteric LN from uninfected or SIV-infected macaques according to the method of Schnizlein et al. (1985) with modifications (Kosco et al., 1992; Rosenberg et al. 1997). Cultured cells were analyzed phenotypically by FCM and for their proliferative ability by the incorporation of [$^3$H]-TdR following a 6-8-hr pulse. In some cases, 1-ml cultures ($2.5 \times 10^6$ cells/well) were set up and 100-$\mu$l aliquots from each of three wells were pulsed with [$^3$H]-TdR before harvesting producing similar results. Other control cultures were comprised of single-cell suspensions produced by passing a portion of the same unfractionated LN through a sieve; such mechanical disruption results in the destruction of the FDC (Schnizlein et al., 1985). Although the latter controls may be suboptimal due to a loss of plasma cells and blasts noted using this method, the rapid loss of LN cells with high-background incorporation in some of these FDC-deprived cultures served to indicate the important role of FDC in sustaining proliferation.

### Immunoglobulin and Anti-SIV Antibody Assays

IgG1 levels and anti-SIV antibody levels were measured using commercial ELISA kits, which cross react with macaque immunoglobulin obtained from The Binding Site (Birmingham, UK) and Genetic Systems (Seattle, WA).

### *Acknowledgements*

The authors wish to thank Drs. Polly Matzinger and Dennis Klinman for helpful comments and Deborah Joynes for her work on the illustration. The project or effort depicted was sponsored in part by the Department of the Army, DAMD17-93-V-3004, and the content of the information does not necessarily reflect the position or the policy of the government and no official endorsement should be inferred.

### *References*

Adams D.H., Shaw S., and van Seventer G. (1994). Lymphocyte adhesion molecules: Role in cell adhesion and intercellular communication. In Handbook of B and T Lymphocytes, Snow, E.C., Ed. (New York: Academic Press), pp. 3-25.

Amadori A., and Chieco-Bianchi L. (1990). B cell activation and HIV-1 infection: Deeds and misdeeds. Immunol. Today **11**: 374-379.

Amadori A., Zamarchi R., Ciminale V., Del Mistro A., Siervo S., Alberti A., Colombatti M., and Chieco-Bianchi L. (1989). HIV-1-specific B cell activation: A major constituent of spontaneous B cell activation during HIV-1 infection. J. Immunol. **143**: 2146-2152.

Amadori A., Zamarchi R., Veronese M.L., Panozzo M., Barelli A., Borri A., Sironi M., Colotta F., Mantovani A., and Chieco-Bianchi L. (1991). B cell activation during HIV-1 infection. II. Cell-to-cell interactions and cytokine requirement. J. Immunol. **146**: 57-62.

Anderson A.O., and Reynolds J.A. (1979). Adjuvant effects of the lipid amine CP-20,961. J. Reticuloendo. Soc. **26**: 667-680.

Apel M., and Berek C. (1990). Somatic mutations in antibodies expressed by germinal center B cells early after primary immunization. Int. Immunol. **2**: 813-819.

Arthur L.O., Bess J.W. Jr., Sowder R.C. II, Benveniste R.E., Mann D.L., Chermann J.-C., and Henderson L.E. (1992). Cellular proteins bound to immunodeficiency viruses: Implications for pathogenesis and vaccines. Science **258**: 1935-1938.

Biberfeld P., Chayt K.J., Marselle L.M., Biberfeld G., Gallo R.C., and Harper M.E. (1986). HTLV-III expression in infected lymph nodes and relevance to pathogenesis of lymphadenopathy. Am. J. Pathol. **125**: 436-442.

Binley J., Cao Y., Jones I., Conner R., Ho D.D., and Moore J.P. (1997). Differential regulation of the antibody response to gag and env proteins of human immunodeficiency vitus type 1. AIDS Res. Human Retrovir. **71**: 2779-2785.

Birx D.L., Lewis M.G., Vahey M., Tencer K., Zack P.M., Brown C.R., Jahrling P.B., Tosato G., Burke D., and Redfield R. (1993). Association of interleukin 6 in the pathogenesis of acutely fatal $SIV_{smm/PBj-14}$ in pigtailed macaques. AIDS Res. Human Retrovir. **9**: 1123-1129.

Bonnefoy J.Y., Henchoz S., Hardie D., Holder M.J., and Gordon J. (1993). A subset of anti-CD21 antibodies promote the rescue of germinal center B cells from apoptosis. Eur. J. Immunol. **23**: 969-972.

Burton G.F., Conrad D.H., Szakal A.K., and Tew J.G. (1993). Follicular dendritic cells and B cell costimulation. J. Immunol. **150**: 31-38.

Chirmule N., Kalyanaraman V.S., Lederman S., Oyaizu N., Yagura H., Yellin M.J., Chess L., and Pahwa S. (1993). HIV-gp160-induced T cell-dependent B cell differentiation: Role of T cell-B cell activation molecule and IL-6. J. Immunol. **150**: 2478-2486.

Colditz I.G., and Watson D.L. (1992). The effects of cytokines and chemotactic agonists on the migration of T lymphocytes into skin. Immunology **76**: 272-282.

Cosentino M.L., Leitman S.F., and Klinman D.M. (1994). Human immunodeficiency virus-specific B cells localize to the bone marrow of gp 160-immunized mice and humans. Vaccine Res. **3**: 195-202.

Delfraissy J.-F., Wallon C., Boue F., Goujard C., Barre-Sinoussi F., and Galanaud P. (1992). HIV-induced, HIV-specific *in vitro* antibody response by B-cells from HIV-seropositive individuals. AIDS **6**: 55-63.

Devergne O., Peuchmaur M., Crevon M.-C., Trapani J.A., Maillot M.-C., Galanaud P., and Emilie D. (1991). Activation of cytotoxic cells in hyperplastic lymph nodes from HIV-infected patients. AIDS **5**: 1071-1079.

Ebnet K., Kaldjian E.P., Anderson A.O., and Shaw S. (1996). Orchestrated information transfer underlying leukocyte endothelial interactions. Ann. Rev. Immunol. **14**: 155-177.

Edelman A.S., and Zolla-Pazner S. (1991). Proliferative response of mononuclear cells from HIV-infected patients to B-cell mitogens: Effects of lymphocyte subset frequency, T-cells defects and prostaglandins. AIDS Res. Human Retrovir. **7**: 953-961.

Emilie D., Peuchmaur M., Maillot M.C., Crevon M.C., Brousse N., Delfraissy J.F., and Dormont J. (1990). Production of interleukins in human immunodeficiency virus-1-replicating lymph nodes. J. Clin. Invest. **86**: 148-159.

Eriksson K., Kilander A., Hagberg L., Norkrans G., Holmgren J., and Czerkinsky C. (1995). Virus-specific antibody production and polyclonal B-cell activation in the intestinal mucosa of HIV-infected individuals. AIDS **9**: 695-700.

Fan J., Bass H.Z., and Fahey J.L. (1993). Elevated IFN-$\gamma$ and decreased IL-2 gene expression are associated with HIV infection. J. Immunol. **151**: 5031-5040.

Fenouillet E., Blanes N., Coutellier A., Demarquest J., Rozenbaum W., and Gluckman J.C. (1992). Monitoring of antibodies against human immunodeficiency virus type 1 p25 core protein as prognostic marker. J. Infect. Dis. **166**: 611-616.

Garrone P., Neidhardt E.M., Garcia E., Galibert L., van Kooten C., and Banchereau J. (1995). Fas ligation induces apoptosis of CD40-activated human B lymphocytes. J. Exp. Med. **182**: 1265-1273.

Graziosi C., Pantaleo G., Gantt K.R., Fortin J.-P., Demarest J.F., Cohen O.J., Sekaly R.P., and Fauci A.S., (1994). Lack of evidence for the dichotomy of $T_H1$ and $T_H2$ predominance in HIV-infected individuals. Science **265**: 248-252.

Hirsch V.M., Zack P.M., Vogel A.P., and Johnson P.R., (1991). Simian immunodeficiency virus infection of macaques: End-stage disease is characterized by widespread distribution of proviral DNA in tissues. J. Infect. Dis. **163**: 976-988.

Jacob J., Kelsoe G., Rajewsky K., and Weiss U., (1991). Intraclonal generation of antibody mutants in germinal centres. Nature **354**: 389-392.

Janossy G., Bofill M., Johnson M., and Racz P., (1991). Changes of germinal center organization in HIV-1-positive lymph nodes. In *Accessory Cells in HIV and Other Retroviral Infections*, Racz, P., Dijkstra, C.D., and Gluckman, J.C., Eds. (Hamburg: Karger), 111-123.

Joling P., Van Wichen D.F., Parmentier H.K., Biberfeld P., Bottiger D., Tschopp J., Rademakers L.H.P.M., and Schuurman H.-J., (1992). Simian immunodeficiency virus ($SIV_{sm}$) infection of cynomolgus monkeys: Effects on follicular dendritic cells in lymphoid tissue. AIDS Res. Human Retrovir. **8**: 2021-2030.

Klaus G.G.B., Humphrey J.H., Kunkl A., and Dongworth D.W., (1980). The follicular dendritic cell: Its role in antigen presentation in the generation of immunological memory. Immunol. Rev. **53**: 3-28.

Koopman G., Parmenter H.K., Schuurman H.-J., Newman W., Meijer C.J.L.M., and Pals S.T., (1991). Adhesion of human B cells to follicular dendritic cells involves both the lymphocyte function-associated antigen 1/intercellular adhesion molecule 1 and very late antigen 4/vascular cell adhesion molecule 1 pathways. J. Exp. Med. **173**: 1297-1304.

Kosco M.H., Pflugfelder E., and Gray D., (1992). Follicular dendritic cell-dependent adhesion and proliferation of B cells in vitro. J. Immunol. **148**: 2331-2339.

Kosco-Vilbois M.H., Gray D., Scheidegger D., and Julius M., (1993). Follicular dendritic cells help resting B cells to become effective antigen-presenting cells: Induction of B7/BB1 and upregulation of major histocompatibility complex class II molecules. J. Exp. Med. **178**: 2055-2066.

Kraal G., Weissman I.L., and Butcher E.C., (1982). Germinal centre B cells: Antigen specificity and changes in heavy chain expression. Nature **298**: 377-379.

Laman J.D., Claassen E., Van Rooijen N., and Boersma W.J.A., (1989). Immune complexes on follicular dendritic cells as a target for cytolytic cells in AIDS. AIDS **3**: 543-544.

Le Hir M., Bluethmann H., Kosco-Vilbois M.H., Muller M., di Padova F., Moore M., Ryffel B., and Eugster H-P., (1996). Differentiation of follicular dendritic cells (FDC) and full antibody responses require TNF receptor-1 (TNFR1) signalling. J. Exp. Med. **183**: 2367-2373.

Liu Y-J., and Banchereau J., (1996). The paths and molecular controls of peripheral B-cell development. The Immunologist **4**: 55-66.

Liu Y.J., Johnson G.D., Gordon J., and MacLennan I.C., (1992). Germinal centres in T-cell-dependent antibody responses. Immunol. Today **13**: 17-21.

Liu Y.J., Mason D.Y., Johnson G.D., Abbot S., Gregory C.D., Hardie D.L., Gordon J., and MacLennan I.C., (1991). Germinal center cells express bcl-2 protein after activation by signals which prevent their entry into apoptosis. Eur. J. Immunol. **21**: 1905-1910.

Lub M., van Kooyk Y., and Figdor C.G., (1995). Ins and outs of LFA-1. Immunol. Today **16**: 479-483.

Macchia D., Almerigogna F., Parronchi P., Ravina A., Maggi E., and Romagnani S. (1993). Membrane tumour necrosis factor-a is involved in the polyclonal B-cell activation induced by HIV-infected human T cells. Nature **363**: 464-466.

Mackay C.R. (1991). T cell memory: The connection between function, phenotype and migration pathways. Immunol. Today **12**: 189-192.

MacLennan I.C.M., and Gray D. (1986). Antigen-driven selection of virgin and memory B cells. Immunol. Rev. **91**: 61-85.

Maeda K., Burton G.F., Padgett D.A., Conrad D.H., Huff T.F., Masuda A., Szakal A.K., and Tew J.G. (1992). Murine follicular dendritic cells and low affinity Fc receptors for IgE (FceRII). J. Immunol. **148**: 2340-2347.

Marschang P., Gurtler L., Totsch M., Thielens N.M., Arlaud G.J., Hittmair A., Katinger H., and Dierich M.P. (1993). HIV-1 and HIV-2 isolates differ in their ability to activate the complement system on the surface of infected cells. AIDS **7**: 903-910.

Masuda A., Burton G.F., Szakal A.K., and Tew J.G. (1995). Loss of follicular dendritic cells in murine-acquired immunodeficiency syndrome. Lab. Invest. **73**: 511-520.

Matsumoto M., Mariathasan S., Nahm M.H., Baranyay F., Peschon J.J., and Chaplin D.D. (1996). Role of lymphotoxin and the type I TNF receptor in the formation of germinal centers. Science **271**: 1289-1291.

May M.J., and Ager A. (1992). ICAM-1-independent lymphocyte transmigration across high endothelium: Differential up-regulation by interferon-gamma, tumout necrosis factor-alpha and interleukin 1 beta. Eur. J. Immunol. **22**: 219-226.

Meerloo T., Sheikh M.A., Bloem A.C., De Ronde A., Schutten M. Van Els C.A.C., Roholl P.J.M., Joling P., Goudsmit J., and Schuurman H.-J. (1993). Host cell membrane proteins on human immunodeficiency virus type 1 after *in vitro* infection of H9 cells and blood mononuclear cells. An immuno-electron microscopic study. J. Gen. Virol. **74**: 129-135.

Mizuma H., Litwun S., and Zolla-Pazner S. (1988). B cell activation in HIV infection: Relationship of spontaneous immunoglobin secretion to various immunological parameters. Clin. Exp. Immunol. **71**: 410-416.

Montaner L.J., (1994). Understanding HIV1, cytokines and effective immunity: Bridging the gap between cytokine regulation *in vitro* and *in vivo*. Res. Immunol. **145**: 575-577.

Persidsky Y., Steffan A.M., Gendrault J.L., Royer C., Beyer C., Muchmore E., Kirn A., and Aubertin A.M. (1995). Morphological changes in lymph nodes and expression of VCAM1 and cytokines at the late stages of SIV-induced disease in rhesus monkeys. Res. Virol. **146**: 185-200.

Petrasch S.G., Kosco M.H., Perez-Alvarez C.J., Schmitz J., and Brittinger G. (1991). Proliferation of germinal center B lymphocytes *in vitro* by direct membrane contact with follicular dendritic cells. Immunobiology **183**: 451-462.

Racz P., Tenner-Racz K., Kahl C., Feller A.C., Kern P., and Dietrich M. (1986). Spectrum of morphological changes of lymph nodes from patients with AIDS or AIDS-related complex. Prog. Allergy **37**: 81-181.

Rideout B.A., Lowenstine L.J., Hutson C.A., Moore P.F., and Pedersen N.C. (1992). Characterization of morphologic changes and lymphocyte subset distribution in lymph nodes from cats with naturally acquired feline immunodeficiency virus infection. Vet. Pathol. **29**: 391-399.

Rosenberg Y.J., Lewis M.G., Villinger F., and Ansari A.A. (1994a). Cytokines and simian immunodeficiency virus infections. Res. Immunol. **145**: 706-713.

Rosenberg Y.J., Zack P.M., Leon E.C., White B.D., Papermaster S.F., Hall E., Greenhouse J.J., Eddy G.A., and Lewis M.G. (1994b). Immunological and virological changes asociated with the decline in the CD4/CD8 ratios in lymphoid organs of SIV infected macaques. AIDS Res. Human. Retrovir. **19**: 863-872.

Rosenberg Y.J., Lewis M.G., Greenhouse J.J., Cafaro A., Leon E.C., Brown C.R., Bieg K.E. and Kosco-Vilbois M.H. (1997). Enhanced follicular dendritic cell functions in lymph nodes of similar immunodeficiency virus-infected macaques: consequences for pathogenesis. Eur. J. Immunol. (in press).

Ruco L.P., Pomponi D., Pigott R., Gearing A.J., Baiocchini A., and Baroni C.D. (1992). Expression and cell distribution of the intercellular adhesion molecule, vascular cell adhesion molecule, endothelial leukocyte adhesion molecule, and endothelial cell adhesion molecule (CD31) in reactive human lymph nodes and in Hodgkin's disease. Amer. J. Pathol. **140**: 1337-1344.

Scala G., Ruocco M.R., Ambrosino C., Mallardo M., Giordano V., Baldassarre F., and Dragonetti E. (1994). The expression of the Interleukin 6 gene is induced by the human immunodeficiency virus 1 TAT protein. J. Exp. Med. **179**: 961-971.

Schnizlein C.T., Kosco M.H., Szakal A.K., and Tew J.G. (1985). Follicular dendritic cells in suspension: Identification, enrichment, and initial characterization indicating immune complex trapping and lack of adherence and phagocytic activity. J. Immunol. **134**: 1360.

Schwartz D.H., Cosentino L.M., Shirai A., Conover J., Daniel S., and Klinman D.M. (1994). Lack of correlation between the number of circulating B cells and the concentration of serum antibodies reactive with the HIV-1 envelope glycoprotein. J. Acquir. Immune Defic. Syndr. **7**: 447-453.

Shirai A., Cosentino M., Leitman-Klinman S.F., and Klinman D.M. (1992). Human immunodeficiency virus infection induces both polyclonal and virus-specific B cell activation. J. Clin. Invest. **89**: 561-566.

Tenner-Racz K., Racz P., Dietrich M., Kern P., Janossy G., Veronese-Dimarzo F., Klatzmann D., Gluckman J.-C., and Popovic M. (1987). Monoclonal antibodies to human immunodeficiency virus: Their relation to the patterns of lymph node changes in persistent generalized lymphadenopathy and AIDS. AIDS **1**: 95-104.

Tenner-Racz K., Racz P., Thome C., Meyer C.G., Anderson P.J., Schlossman S.F., and Letvin N.L. (1993). Cytotoxic effector cell granules recognized by the monoclonal antibody TIA-1 present in CD8+ lymphocytes in lymph nodes of human immunodeficiency virus-1 infected patients. Amer. J. Pathol. **142**: 1750-1758.

Tew J.G., Kosca M.H., Burton G.F., and Szakal A.K. (1992). Follicular dendritic cells as accessory cells. Immunol. Rev. **117**: 185-211.

Thielens N.M., Bally I.M., Ebenbichler C.F., Dierich M.P., and Arlaud G.J. (1993). Further characterization of the interaction between the C1q subcomponent of human C1 and the transmembrane envelope glycoprotein gp41 of HIV-1. J. Immunology **151**: 6583-6592.

Vago L., Antonacci C., Cristina S., Parravicini C., Lazzarin A., Moroni M., Negri C., Uberti-Foppa C., Musicco M., and Costanzi G. (1989). Morphogenesis, evolution and prognostic significance of lymphatic tissue lesions in HIV infection. Appl. Pathol. **7**: 298-309.

Virelizier J.L. (1994). How HIV may escape the activating effects of TNF. Res. Immunol. **145**: 690-696.

Westermann J., and Pabst R. (1992). Distribution of lymphocyte subsets and natural killer cells in the human body. Clin. Invest. **70**: 539-544.

Yarchoan R., Redfield R.R., and Broder S. (1986). Mechanisms of B cell activation in patients with acquired immunodeficiency syndrome and related disorders. Contribution of antibody-producing B cells, of Epstein-Barr virus-infected B cells, and of immunoglobulin production induced by human T cell lymphotropic virus, type III/lymphadenopathy-associated virus. J. Clin. Invest. **78**: 439-447.

Zolla-Pazner S. (1984). B cells in the pathogenesis of AIDS. Immunol. Today **5**: 289-291.

